# Parental Support and Adolescents’ Coping with Academic Stressors: A Longitudinal Study of Parents’ Influence Beyond Academic Pressure and Achievement

**DOI:** 10.1007/s10964-023-01864-w

**Published:** 2023-09-21

**Authors:** Melanie J. Zimmer-Gembeck, Ellen A. Skinner, Riley A. Scott, Katherine M. Ryan, Tanya Hawes, Alex A. Gardner, Amanda L. Duffy

**Affiliations:** 1 School of Applied Psychology and Griffith Centre for Mental Health, Griffith University, Gold Coast, QLD Australia; 2 Portland State University, Portland, OR USA; 3 School of Applied Psychology, Griffith University, Gold Coast, QLD Australia

**Keywords:** Academic stress, Academic achievement, Coping, Parenting, Parental support

## Abstract

Adolescents face many academic pressures that require good coping skills, but coping skills can also depend on social resources, such as parental support and fewer negative interactions. The aim of this study was to determine if parental support and parental negative interactions concurrently and longitudinally relate to adolescents’ ways of academic coping, above and beyond the impact of three types of academic stress, students’ achievement at school (i.e., grades in school), and age. Survey data were collected from 839 Australian students in grades 5 to 10 (*M*_age_ = 12.2, *SD* = 1.72; 50% girls). Students completed measures of support and negative interactions with parents; academic stress from workload, external pressure (teachers/parents) to achieve, and intrapsychic pressure for high achievement; and ways of academic coping that were grouped into two positive and two negative types. Hypothesized associations were tested concurrently and from one year to the next using path modeling. Beyond the numerous significant influences of academic stress and achievement on coping, and control for age and COVID-19 timing, adolescents with more parental support reported more use of engagement coping (e.g., strategizing) and comfort-seeking, whereas those who reported more negative interactions with parents reported more use of disengagement coping (e.g., concealment) and escape. In the longitudinal model, parental support predicted an increase in engagement and comfort-seeking and a decrease in disengagement coping, whereas negative interaction with parents predicted an increase in disengagement coping. Overall, the findings support the view that coping with academic stressors will continue to depend on parent-adolescent relationships even into the teen years.

## Introduction

For adolescents around the world, academic workload and other school-related demands are stressors that prompt many coping responses (Raftery-Helmer & Grolnick, 2015). Without adequate coping, academic stressors can have a cumulative negative effect and substantially interfere with motivation, engagement, and optimal learning, change future opportunities (Skinner & Saxton, 2019), and contribute to personal distress and psychological disorders (Schönfeld et al., 2019). Furthermore, academic stressors can occur for many different reasons. Some adolescents report workloads that overwhelm their abilities and their time but, for others, stressors are more internal or intrapsychic and relate to high self-expectations of achievement and pressure to be the very best (Sun et al., 2011). Another source of stress can be external, with parents and teachers directly communicating that adolescents could and should do better academically. In fact, these are the three most common reasons for academic stress: a perceived high level of schoolwork (*workload pressure*), an internal drive for high achievement (*intrapsychic pressure*), and external pressure to achieve from parents or teachers (*external pressure*; Bjorkman, 2007). The recognition that adequate coping is needed to overcome these forms of academic stress, and that stressful events and coping can affect adolescents’ development and well-being, has led to a great deal of research identifying how teachers (Raftery & Grolnick, 2018) and the classroom environment (Shih, 2015) can support adolescents’ coping. Yet, parent-adolescent relationships have also been linked to adolescents’ academic ways of coping (Zimmer-Gembeck & Locke, 2007), but no previous research has considered how support *and* negative interactions with parents may uniquely account for adolescents’ ways of coping with the multitude of academic stressors they can encounter (Skinner & Saxton, 2019). To fill this gap, the roles of parental support and parent-adolescent negative interactions in adolescents’ engagement and disengagement ways of coping with workload, intrapsychic, and external sources of academic stress were investigated in the current study.

### Academic Stressors and Ways of Coping: Engagement and Disengagement Coping

Regardless of the source of academic stress, adolescents rely on a range of ways of coping in response (Morales-Castillo, 2022). Academic coping includes the many ways that students respond when they face academic challenges, setbacks, and difficulties (Skinner et al., 2013). To develop a specific understanding of the different ways students can cope, scholars interested in academic coping have relied on the numerous categorizations of coping that have been developed across decades of research on stress and coping (e.g., Skinner & Saxton, 2019). One conceptualization that has been very useful for understanding child and adolescent achievement and well-being has been the differentiation of engagement from disengagement ways of coping (Conner-Smith et al., 2000). *Engagement* includes coping responses that orient towards the stressor to tackle it more directly or to engage others in providing support. *Disengagement* coping encompasses responses that involve turning away from active attempts to modify the stressful event or reduce distress – sometimes even prompting more distress through excessive worry or self-blame. Within an academic context, engagement coping has been shown to be most adaptive for promoting academic achievement, participation, and tenacity. Students who approach and engage with challenges to learn, achieve better grades, and are more behaviorally involved and emotionally positive about school (Skinner et al., 2020). For example, engagement forms of coping, such as strategizing and seeking information, have been found to reduce future stress and have been positively related to intrinsic interest in learning (Appelhans & Schmeck, 2002). Conversely, in this same study, disengagement ways of coping (e.g., concealing problems, ruminative thoughts about workload or achievement pressures, or minimizing the importance of schoolwork) were related to lower academic performance.

Engagement and disengagement ways of coping can follow from adolescents’ experiences of academic stress from workload, intrapsychic expectations for achievement, and/or external pressures (Morales-Castillo, 2022). Engagement ways of coping encompassed some of the most active approach responses appropriate for academic stressors, namely strategizing, help-seeking, comfort-seeking, self-encouragement, and commitment to the task or goal. Disengagement ways of coping, which align with avoidance or nonproductive forms of coping, were measured as confusion, concealment, self-pity, rumination, and escape. Taken together, these ways of coping with academic stressors capture the range of strategies that adolescents report relying on to manage their emotions and motivations related to academic pressures, to improve (or worsen) the stressful situation, and to put in place plans or solve problems in ways that can reduce (or worsen) the likelihood of academic stressors becoming chronic and impairing (Skinner et al., 2016).

Multiple strands of research provide evidence supporting the focus on this range of academic coping responses. In this past research, student well-being, motivation, participation, and achievement have been found to be associated with engagement coping (Shih, 2015; Wang & Eccles, 2012). Other past research identified concealment (possibly the antithesis of help-seeking) as blocking participation and learning (Ryan et al., 2005), and escape, withdrawal, helplessness, and rumination as indicators of avoidance of tackling academic challenges (Skinner et al., 2016; Vizoso et al., 2019). This research has found that these disengagement ways of coping make academic participation and achievement more difficult, and they relate to increased distress, burnout, and the likelihood of giving up. Although coping is sometimes considered to be an individual affair, many of these coping responses include the involvement of other people, for example, in providing opportunities for help and comfort when it is sought or in constraining opportunities for assistance that might make escape, withdrawal, and concealment more likely. This fits with decades of research indicating that the availability of social resources can impact stress and coping (Lazarus & Folkman, 1984). When it comes to schoolwork, some of the most important social resources for academic stressors can be found in relationships with parents.

### Academic Coping as Related to Adolescents’ Perceived Parenting Experiences

Parents have been frequently described as a primary source of modeling and socialization of their children’s development of coping (e.g., see Skinner & Zimmer-Gembeck, 2016 for a review). The recognition of the role of family in learning about (and the development of) coping has led those with a developmental view of stress and coping to encourage a greater focus on identifying the social foundations of coping itself (Skinner and Edge 2002a). For example, in a review, Compas et al. (2001) proposed that researchers “need to pay closer attention to the social context in which children encounter and try to cope with stress” (p. 122). Given the recognition of the importance of this topic, there has been research on the teacher-relationships and school contexts that assist adolescents to better cope with academic stress (Raftery-Helmer & Grolnick, 2018), but there not been much attention on parent-adolescent relationships. For example, in a recent review of studies of academic coping, only 16 of the 66 reviewed studies considered social antecedents, and, of these, most considered teachers and classroom contexts (Skinner & Saxton, 2019).

The idea that parent-adolescent relationships should be social foundations for adolescents’ academic coping is supported by self-determination theory (SDT; Deci & Ryan, 1985), which has suggested that when parents meet child and adolescent needs for relatedness, competence, and autonomy, this encourages their engagement, and minimizes their disengagement, ways of coping with stressors (Ntoumanis et al., 2009; Skinner & Edge, 2002b). Parents can support adolescents’ psychological needs for relatedness, competence, and autonomy through the provision of support and involvement, encouragement, and communication and feedback about progress in and outside of school, and by using autonomy supportive strategies to encourage choice, participation, and internalized motivation for success (Klootwijk et al., 2022). Central to an SDT-influenced motivational theory of the development of coping is the understanding that attachment (communication and trust in the parent-adolescent relationship), and, conversely, experiences of coercion and rejection in important social relationships will influence whether coping or patterns of action when facing stress will involve engagement or disengagement (Skinner & Wellborn, 1994). Thus, social environments that include relations that are connected and warm are expected to promote positive, active, and engaged coping behaviors. Social environments that include relationships with others that are hostile, rejecting, and coercive will yield unproductive, avoidant, and disengaged or helpless coping responses.

The theoretical ways that parents may influence their children’s coping are wide-ranging and include coaching and modeling, the quality of the parent-child relationship, the family environment, and family structure (Power, 2004). Of these influences, general parental support versus rejection and coercion are the aspects of parenting that may most directly spill over into academic stress and coping. For children and adolescents, good communication and trust in the support of a parent are closely connected to coping responses and, as outlined in detail in attachment theory (Zimmer-Gembeck et al., 2017), adolescents’ and adults’ coping can be more productive when there is just the possibility that positive support is available (for example, the belief that talking to a parent is possible). Thus, by adolescence, perceived availability of parental support would be expected to be a resource for greater action and more engaged coping responses to academic stress. Conversely, if parents are perceived as unsupportive, rejecting, hostile, and coercive, this might translate to unproductive responses when adolescents are coping with academic stressors. In one of the only studies to examine these relations, cohesive, low conflict, communicative families were more likely to model active coping behaviors for children, and they had children who more frequently used active coping behaviors and exhibited fewer problematic responses when dealing with stressful events (Kliewer et al., 1996). In a second study, adolescents who reported more involved and autonomy supportive parents used more engagement (i.e., active) coping with problems at home and at school (Zimmer-Gembeck & Locke, 2007). Although no previous study was found that had examined whether parental support and negative interactions between parents and adolescents are associated with adolescents’ engagement and disengagement coping with academic stress, one study of 183 young adolescents reported that parental involvement was associated with more mastery academic coping (i.e., problem-solving, help-seeking, and support for feelings), but not associated with defensive coping (i.e., rumination and blame) after receiving a bad grade (Raftery-Helmer & Grolnick, 2018).

### Age

The early to middle adolescent years bring change in academics, relationships, stressors, and skills at coping. This age period is when parent-child relationships may become more negative in their interactions (Branje, 2018) as adolescents desire more autonomy and parents are adjusting to these changes (Zimmer-Gembeck et al., 2011). In addition, from early to middle adolescence, academic demands and external pressures can increase (Seiffge-Krenke et al., 2009), and there is evidence that engagement may decrease and disengagement coping may increase with age (Ben-Eliyahu & Kaplan, 2015) alongside a general increase in school demands and decrease in connection to school (Skinner & Saxton, 2019). Thus, in general, age-related changes have been found in academic stress level, ways of coping, and negative interactions with parents across the early to middle adolescent years. All such adolescent (as well as family and school-related) changes suggest that age should be accounted for when studying relations between parent-adolescent relationships, academic stressors, and ways of academic coping.

## Current Study

Parents are known to model, encourage, teach, and support adolescents’ ways of coping with stress. Yet, much of the research on social foundations of academic coping has concentrated on teachers and school, with very little research on the implications of parent-adolescent relationships for adolescents’ academic coping, especially when consider stress from workload, and intrapsychic and external pressure to achieve. The aim of this study was to test concurrent and longitudinal associations of parental support and adolescents’ reports of their negative interaction with their parents (i.e., experiences of rejection and coercion) with adolescents’ engagement and disengagement ways of coping with academic stress in the context of workload, as well as intrapsychic and external, pressures. There were two hypotheses. First, adolescents’ perceptions of parental support will relate to more engagement and less disengagement academic coping, both concurrently and by the next year, above and beyond the impacts of workload, intrapsychic and external stress, achievement, and age (Hypothesis 1). Second, negative interactions with parents will have the opposite associations, relating to less engagement and more disengagement academic coping, both concurrently and by the next year (Hypothesis 2). While testing these two hypotheses, the relations of three types of stressors, namely workload, intrapsychic, and external pressures, as well as adolescents’ achievement (measured as “usual” grades in school) and age, were considered as additional correlates of adolescents’ ways of coping. Finally, T1 data were collected before, during, and after a major stay-at-home order (SAHO) for COVID-19 in Australia. Thus, differences between these three groups of students were described and COVID-19 timing of data collection was included in the primary models.

## Methods

### Participants

The participants were 839 Australian students in grades 5 to 10 who participated in T1 of a 1-year longitudinal study (two waves of data collection). The number of students who attempted the survey at T1 was 882, but 22 participants were excluded because of patterned responding and 21 were excluded because they did not complete more than the first measure. Of the 839 remaining adolescent participants, 96% were aged 10 to 15 years (1% were age 9 and 3% were age 16 or 17; *M*_age_ = 12.2, *SD* = 1.72), 49% reported boys, 50% girls, and 1% nonbinary/other. Adolescents could report race/ethnicity and/or Australia or New Zealand as their birth country; 47% reported White; 6% Asian; 4% Australian First Peoples, Torres Strait Islander or Pacific Islander; and 29% other (reporting more than 20 different backgrounds). The remaining 20% did not tick any race/ethnicity. More than one-half (56%) reported they were born in Australia and 6% were born in New Zealand.

### Procedure

Following approval of the study by the Griffith University human research ethics committee (Reference #2019/178) and the Queensland (Australia) state education department, local schools were provided information about the study via email and telephone. The first three consenting secondary schools were included in the study and, subsequently, their feeder primary schools were invited to participate, for a total of eight participating schools (in Queensland Australia, students attend primary school until grade 6 and then transition to secondary school for grades 7 to 12). The schools attracted students across all income brackets. Depending on the school, 14–29% of the student population was within the lowest income quartile, and 4–30% was within the highest income quartile.

To gather informed consent from parents, students took consent forms home for completion and returned them to the school. Across the schools, 52% of students returned consent forms to the school and, of these, 80% of parents gave informed consent for participation. All consent processes were conducted in the schools in 2019 and 2020, prior to a national COVID-19 pandemic SAHO that continued for about one month for primary and secondary schools. In 2019 prior to SAHO, T1 questionnaires were completed by 350 students in their regular classrooms. However, in 2020, data were collected from 240 students while under SAHO (but school continued online). The remaining 249 students completed the questionnaire online from home in 2020 after classroom teaching started again, but schools did not allow researchers to attend in person. These three groups of students were compared, and COVID-related timing of survey completion was included as a covariate in all analyses.

The portions of the survey included in this study were completed in approximately 20 min at each of T1 and one year later at T2. The entire survey was focused on relationships, stress, and student well-being. Other measures included in the survey but not analyzed here concerned additional stressful events and coping with these events (peer relationships and world or community crises), friendship support, and emotional problems. At T1, each student who participated at school prior to COVID-19 SAHO received a small gift for their participation, whereas others who completed the survey online from home or after SAHO received a $20 gift voucher. At T2, each student who completed the survey received a $20 gift voucher.

### Measures

#### Academic Coping

At T1 and T2, 10 ways of coping with academic stressors were measured with two items each drawn from the measure of Coping Reactions to School Challenges (20 items total; Skinner et al., 2013). Five ways of engaged coping were measured, including strategizing (“I think of some things that will help me next time”, *r* = 0.53), help-seeking (“I get some help on the parts I didn’t understand”, *r* = 0.61), comfort-seeking (“I talk about it with someone who will make me feel better”, *r* = 0.57), self-encouragement (“I tell myself I’ll do better next time”, *r* = 0.53), and commitment (“I remind myself that it’s something I really want to do”, *r* = 0.49). Five ways of disengaged coping were measured, including confusion (“It’s difficult for me to think”, *r* = 0.45), rumination (“feel like you can’t get it out of your head”, *r* = 0.57), concealment (“I try to hide it”, *r* = 0.58), self-pity (“I say ‘This always happens to me’”, *r* = 0.43), and escape (indicative of minimization of the stressor; e.g., “say it wasn’t important”, *r* = 0.50). Prior to responding to coping items, students were asked “When something bad happens in your schoolwork (like not doing well on a test or not being able to answer an important question), or you are having trouble with a subject at school, how much do you…”. Responses for each coping item ranged from 1 (*I don’t do this at all or I do this a little*) to 4 (*So much! I do this almost all of the time*).

The ways of coping showed intercorrelations with each other that suggested broader composite scores would represent the coping responses, which was supported by exploratory factor analysis (principal axis factoring with varimax rotation). Using T1 measures, the factor analysis suggested three factors based on the criterion of an eigenvalue > 1 (eigenvalues = 2.71, 2.32, 1.07, 45% of the variance in the items). Yet, two items (comfort-seeking and escape) had high and similar strength loadings on all three factors. These two items were removed, and another factor analysis extracted two factors with eigenvalues > 1 (2.54, 2.03), and accounted for 43% of the variance in the items. Factor 1 had high loadings for rumination (0.81), self-pity (0.76), confusion (0.70), and concealment (0.49). Factor 2 had high loadings for strategizing (0.63), commitment (0.60), self-encouragement (0.57), and help-seeking (0.49). The items loading highly on Factor 1 were averaged to form an aggregate coping score referred to as *disengagement coping* (Cronbach’s α = 0.78 and 0.77 at T1 and T2, respectively). The items on Factor 2 were averaged to form an aggregate coping score referred to as *engagement coping* (Cronbach’s α = 0.66 and 0.60 at T1 and T2, respectively). *Comfort-seeking* and *escape* were maintained as separate ways of coping for the analysis. Thus, four ways of coping were considered in the analyses: engagement, disengagement, comfort-seeking, and escape.

#### T1 Parental Support and Parent-Child Negative Interaction

Parental support was measured with the Inventory of Parent and Peer Attachment-Revised (10 items; Armsden & Greenberg, 1987; “I can count on my parents when I need to talk about something important”, ‘My parents understand me”). Responses ranged from 1 (*No! Not at all true for me*) to 6 (*Yes! Totally true for me*). Responses to items were averaged to form a total score of parental support (Cronbach’s α = 0.87), with a higher score indicating more support.

Parent-child negative interaction (i.e., feelings of rejection and coercion) was measured with 9 items from the Parents as Social Context Questionnaire (Skinner et al., 2005; “My parents make me feel like I’m not wanted”). Responses ranged from 1 (*No! Not at all true for me*) to 6 (*Yes! Totally true for me*). Responses were averaged to form a total score of negative interactions with parents with a higher score indicating more negative interactions, Cronbach’s α = 0.90.

#### T1 External and Intrapsychic Academic Pressure and Workload

Twelve items from the Academic Stress Scale (Bjorkman, 2007) were used to measure external pressure from parents and teachers to perform well in school (4 items; “My parents pressure me to get good grades”, “My teachers pressure me to get good grades”), intrapsychic pressures for academic performance (3 items; “I take my schoolwork too seriously”), and workload pressures (5 items; “I have too much homework to do it all well”). Responses ranged from 1 (*No! Not at all true for me*) to 6 (*Yes! Totally true for me*). Responses to items on each subscale were averaged to form total scores for external pressure (Cronbach’s α = 0.83), intrapsychic pressure (Cronbach’s α = 0.74), and workload pressure (Cronbach’s α = 0.89), with higher scores indicating more pressure.

#### T1 Grades in School

Adolescents reported their usual grades in school (“What grades do you usually get at school?”) on a scale from *Mostly A’s* (1) to *D’s and lower* (6). This item was reversed so a higher score indicated higher achievement.

### Data Analyses

After examining means, standard deviations, and correlations between all measures, model testing involved fitting a concurrent model with paths freed from all measures to concurrent (T1) measures of academic coping, and (separately) fitting a longitudinal model with paths freed from all measures (including T1 coping) to T2 coping. In addition, in both models, covariances were freed between the predictor variables and between the coping variables, but those with *p* > 0.10 were trimmed to produce final models. Age and data collection timing (before, during, or after SAHO) were included as covariates in each model. Model fit was determined by the root mean square error of approximation (RMSEA) and the comparative fit index (CFI). RMSEA values below 0.05 are considered good, values between 0.05 and 0.08 are considered indicative of fair fit, and values between 0.08 and 0.10 are considered an indication of mediocre fit (Kaplan, 2000). The CFI is more acceptable as values approach one; values over 0.95 are considered indication of very good model fit (Hu & Bentler, 1999). Chi-square (χ^2^) and associated *p*-value are also reported. Critical ratios were used to determine significance of model paths (*t*-test values above an absolute value of 1.96).

## Results

### Missing Data and Comparisons of Students Retained or Not at T2

Overall, there were minimal missing data at T1, with 91 (11%) students missing 1 to 4 academic stress and/or coping items, and 50 (6%) missing 1 to 3 parenting items. Because there were so few missing items for any participant on any measure, T1 composite scores were formed for all students based on the completed items, providing T1 scores for all 839 students. One year later (T2), students were recontacted either via their schools, email and/or text to give them access to the second survey. In total, 743 students (89%) completed the T2 survey. Three differences were found when T1 measures were compared between students who were or were not retained at T2. Retained students reported slightly more engagement coping (*M* = 2.40, *SD* = 0.57 vs. *M* = 2.27, *SD* = 0.50, *t*(1837) = −2.16, *p* = 0.031), less workload pressure (*M* = 2.70, *SD* = 1.29 vs. *M* = 3.00, *SD* = 1.30, *t*(1837) = 2.13, *p* = 0.033), and better grades (*M* = 3.97, *SD* = 1.25 vs. *M* = 3.58, *SD* = 1.29, *t*(1837) = 2.89, *p* = 0.004). No significant differences were found for other measures, age, or proportion boy/girl. Also, Little MCAR’s test was not significant supporting the conclusion that missing data at T2 were completely at random, χ^2^ (30) = 28.55, *p* = 0.541. Nevertheless, instead of using listwise deletion, all 839 participants were maintained in all analyses using missing data estimation techniques of multiple imputation (in SPSS v.29) for descriptive statistics and correlations, and FIML for path models. Multiple imputation involved producing 20 imputed datasets, and pooled results for descriptive and correlational analyses are reported below.

### Descriptive Statistics and COVID-Related Differences

Table 1 provides a summary of comparisons of the means (*M*s) of all T1 variables among three groups based on data collection procedures at T1, namely, students who participated (1) pre-COVID, (2) during the first major SAHO in the area (slightly before and into April 2020), and (3) after returning to in-school learning. Notably, age differed between groups, with those participating after return to in-class learning significantly older than students in the other two groups. Other than age differences, there were eight differences with significance levels *p* < 0.005 (0.05 adjusted for 11 comparisons) generally favoring students during SAHO, then pre-COVID, then returning to school: During SAHO, parental support and engagement coping were highest, and parent negative interactions, external pressure, workload pressure, and grades in school were lowest. Students who participated in-class (pre-COVID) were also higher in parental support and lower in external pressure, workload, and grades in school than students who participated after returning to in-school learning. Finally, those who participated after returning to in-class learning were lower in disengagement but higher in escape than the other two groups.

**Table 1:** Comparison of Students in the Three T1 Data Collection Groups on all T1 Measures (*N* = 839)

	*M* (*SD*)					
	In class *n* = 350	COVID *n* = 240	Online after *n* = 249	*F*(2836)	*p*	eta^2^ (95% CI)
Parental support	4.43 (0.99)^b^	4.69 (0.95)^c^	3.87 (1.15)^a^	40.38	<0.001	0.09 (0.05–0.13)
Parent negative interactions	2.36 (1.13)^b^	2.12 (0.93)^a^	2.54 (1.30)^b^	8.18	<0.001	0.02 (0.01–0.04)
Workload pressure	2.71 (1.33)^b^	2.50 (1.26)^a^	3.01 (1.22)^c^	9.84	<0.001	0.02 (0.01–0.05)
Intrapsychic pressure	3.27 (1.24)	3.16 (1.34)	3.21 (1.29)	0.49	0.615	0.00 (0.00–0.01)
External pressure	2.71 (1.33)^b^	2.50 (1.26)^a^	3.01 (1.22)^c^	9.84	<0.001	0.07 (0.04–0.10)
Grades in school	3.88 (1.21)^b^	3.72 (1.28)^a^	4.15 (1.26)^c^	7.38	0.001	0.02 (0.00–0.04)
Disengagement coping	2.47 (0.56)^b^	2.39 (0.59)^b^	2.24 (0.53)^a^	12.74	<0.001	0.03 (0.01–0.05)
Engagement coping	2.13 (0.64)^a,b^	2.01 (0.61)^a^	2.21 (0.69)^b^	5.80	0.003	0.01 (0.00–0.03)
Comfort-seeking	2.27 (0.91)	2.16 (0.90)	2.11 (0.87)	2.42	0.090	0.01 (−0.01–0.02)
Escape	1.66 (0.74)^a^	1.63 (0.69)^a^	1.98 (0.81)^b^	17.95	<0.001	0.04 (0.02–0.07)
Age	11.62 (1.43)^a^	11.52 (1.61)^a^	13.67 (1.21)^b^	189.98	<0.001	0.31 (0.26–0.36)

Mean values with different superscripts are significantly different from each other, *p* < 0.05

Adjusted *p* < 0.005 (0.05/10)

### Correlations between Measures

Pearson’s correlations between measures are detailed in Table 2. Most correlations between parenting and ways of coping were in the expected directions – students who experienced more parental support (and less parent negative interactions) also reported more engagement and less disengagement and escape coping, and more comfort-seeking. In addition, T1 ways of coping were associated with at least three of the four other measures at T1. Students experiencing higher workload and external pressure reported less engagement and more disengagement and escape coping; and students with higher grades in school also reported more engagement and less disengagement coping. Exceptions to these patterns were found for adolescents’ intrapsychic pressure, which was correlated with more coping of most kinds (engagement, disengagement, and comfort-seeking), but less escape. Similar correlations were found for T2 ways of coping with other measures, although they tended to be weaker and some were no longer significant. Correlations with age are also provided in Table 1. Older students reported less parental support, more negative interactions, and more external pressure for academic performance. They also reported more intrapsychic pressure, a higher workload, and better grades. For coping, older students reported less engagement coping and more escape.

**Table 2:** Correlations between All Measures (*N* = 839)

		1	2	3	4	5	6	7	8	9
1	Parental support	–								
2	Parent negative interactions	−0.56^***^	–							
3	External pressure	−0.36^***^	0.46^***^	–						
4	Intrapsychic pressure	0.01	0.17^***^	0.22^***^	–					
5	Workload pressure	−0.30^**^	0.38^***^	0.47^***^	0.19^***^	–				
6	Grades in school	0.06	−0.09^**^	−0.15^***^	0.28^***^	−0.29^***^	–			
7	Disengagement coping	−0.27^***^	0.44^***^	0.36^***^	0.32^***^	0.55^***^	−0.16^***^	–		
8	Engagement coping	0.29^***^	−0.09^**^	−0.08^*^	0.34^***^	−0.20^***^	0.16^***^	0.02	–	
9	Comfort-seeking	0.16^***^	0.01	0.02	0.15^***^	0.06	−0.04	0.13^***^	0.41^***^	–
10	Escape	−0.18^***^	0.24^***^	0.27^***^	−0.11^**^	0.36^***^	−0.18^***^	0.32^***^	−0.18^***^	0.08^*^
11	T2 Disengagement coping	−0.21^***^	0.29^***^	0.17^***^	0.20^***^	0.29^***^	−0.07	0.42^***^	−0.01	0.06
12	T2 Engagement coping	0.24^***^	−0.15^***^	−0.14^***^	0.11^**^	−0.23^***^	0.16^***^	−0.18^***^	0.39^***^	0.12^**^
13	T2 Comfort-seeking	0.15^***^	−0.06	−0.03	0.06	−0.06	0.03	−0.09^*^	0.21^***^	0.24^***^
14	T2 Escape	−0.15^***^	0.18^***^	0.17^***^	−0.03	0.20^***^	−0.10^**^	0.17^***^	−0.13^***^	0.06
15	Age	−0.24^***^	0.07^*^	0.23^***^	0.08^*^	0.12^***^	0.16^***^	0.01	−0.09^**^	−0.05
	Mean	4.34	2.35	2.60	3.22	2.74	3.92	2.12	2.38	2.19
	SD	1.08	1.14	1.35	1.28	1.29	1.26	0.65	0.57	0.90

		10	11	12	13	14
1	Parental support					
2	Parent negative interactions					
3	External pressure					
4	Intrapsychic pressure					
5	Workload pressure					
6	Grades in school					
7	Disengagement coping					
8	Engagement coping					
9	Comfort-seeking					
10	Escape	–				
11	T2 Disengagement coping	0.16^***^	–			
12	T2 Engagement coping	−0.19^***^	−0.01	–		
13	T2 Comfort-seeking	−0.04	−0.01	0.45^***^	–	
14	T2 Escape	0.33^***^	0.30^***^	−0.16^***^	0.05	–
15	Age	0.11^**^	0.04	0.00	−0.01	0.16^***^
	Mean	1.74	2.22	2.39	2.25	1.88
	SD	0.76	0.66	0.58	0.84	0.81

All variables were assessed at Time 1, except those labeled as Time 2 (T2)

*p < 0.05; **p < 0.01; ***p < 0.001

### Concurrent Model: Associations of Parents, Pressure, and Achievement on Ways of Coping

**Fig. 1:**
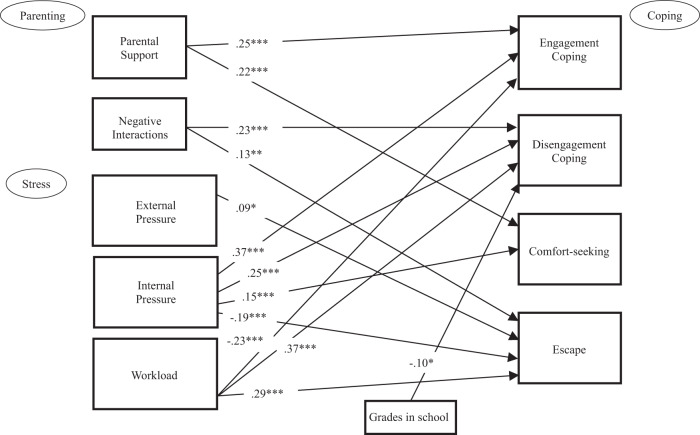
An illustration of the significant path coefficients in the model of concurrent measures of parenting, academic stress, ways of academic coping, and grades. *p < 0.05. **p < 0.01. ***p < 0.001. Adolescent age and COVID-19 data collection timing are not shown here (see Table 3 for results)

The results of the concurrent model linking parental support, parent negative interactions, pressures, and grades in school to the four ways of coping (adjusting for age and data collection timing) are shown in Table 3 and Fig. 1. This model had a good fit to the data, χ^2^(9) = 10.94, *p* = 0.280, CFI = 1.00, RMSEA = 0.016 (90% CI 0.000 to 0.044), *p* = 0.982. Overall, the model accounted for 24% of the variance in academic engagement coping, 42% of disengagement coping, 6% of comfort-seeking, and 21% of escape. As expected, parental support and parent negative interactions were significantly associated with students’ academic coping, even after adjusting for academic pressures, grades in school, age, and data collection timing (before, during SAHO, or after). Parental support was associated with more engaged forms of coping only (β = 0.25 and β = 0.22 for engagement coping and comfort-seeking, respectively, both *p* < 0.001), whereas parent negative interactions was associated with more disengaged forms of coping only (β = 0.23 and β = 0.13 for disengagement coping and escape, respectively, both *p* < 0.001).

**Table 3:** Results of Testing All Directional Paths to Academic Coping in the Concurrent Model (*N* = 839)

Predictor	Outcome	*B*	*SE B*	*p*-value	β
Parental support	Engagement coping	0.13	0.02	<0.001	0.25^***^
Parental support	Disengagement coping	−0.02	0.02	0.278	−0.04
Parental support	Comfort-seeking	0.18	0.04	<0.001	0.22^***^
Parental support	Escape	0.04	0.03	0.162	0.06
Parent neg int	Engagement coping	0.03	0.02	0.125	0.06
Parent neg int	Disengagement coping	0.13	0.02	<0.001	0.23^***^
Parent neg int	Comfort-seeking	0.06	0.03	0.064	0.08
Parent neg int	Escape	0.08	0.03	0.002	0.13^**^
External pressure	Engagement coping	0.01	0.02	0.595	0.02
External pressure	Disengagement coping	0.00	0.02	0.886	0.01
External pressure	Comfort-seeking	−0.01	0.03	0.731	−0.01
External pressure	Escape	0.05	0.02	0.017	0.09^*^
Internal pressure	Engagement coping	0.16	0.02	<0.001	0.37^***^
Internal pressure	Disengagement coping	0.13	0.02	<0.001	0.25^***^
Internal pressure	Comfort-seeking	0.10	0.03	<0.001	0.15^***^
Internal pressure	Escape	−0.11	0.02	<0.001	−0.19^***^
Workload	Engagement coping	−0.10	0.02	<0.001	−0.23^***^
Workload	Disengagement coping	0.19	0.02	<0.001	0.37^***^
Workload	Comfort-seeking	0.04	0.03	0.180	0.06
Workload	Escape	0.17	0.02	<0.001	0.29^***^
Grades in school	Engagement coping	0.00	0.02	0.786	−0.01
Grades in school	Disengagement coping	−0.05	0.02	0.002	−0.10^**^
Grades in school	Comfort-seeking	−0.05	0.03	0.056	−0.07
Grades in school	Escape	−0.02	0.02	0.316	−0.04
Age	Disengagement coping	−0.04	0.01	<0.001	−0.11^**^
Before COVID	Engagement coping	0.12	0.04	0.002	0.11^**^
Before COVID	Disengagement coping	−0.09	0.05	0.062	−0.07
Before COVID	Escape	−0.25	0.06	<0.001	−0.16^***^
After COVID	Engagement coping	0.04	0.05	0.405	0.03
After COVID	Disengagement coping	−0.13	0.05	0.014	−0.09^*^
After COVID	Escape	−0.23	0.07	<0.001	−0.14^***^

*Neg int* negative interactions, Before and After COVID = Dummy coded variables to account for timing of data collection (see Table 1). Covariances among predictors and among coping outcomes were freed if significant at *p* < 0.10. These covariances (and correlations) are not shown in this Table, but they were similar to the results shown in Table 1 and Table 2. Only significant direct paths from age and COVID variables to coping measures were freed

Model fit: χ^2^(9) = 10.94, *p* = 0.280 CFI = 1.00, RMSEA = 0.016 (90% CI 0.000 to 0.044), *p* = 0.982

*p < 0.05; **p < 0.01; ***p < 0.001

As expected, ways of coping were also associated with measures of academic pressures as well as grades in school (see Table 3 and Fig. 1). Students who perceived more external performance pressure from parents and teachers reported more escape (β = 0.09, *p* < 0.05). Students who reported more workload pressure reported less engagement coping (β = −0.23, *p* < 0.001)., and more disengagement and escape (β = 0.37 and β = 0.29, respectively, both *p* < 0.001). Intrapsychic pressure had a more mixed pattern of associations with academic coping, with students reporting higher intrapsychic pressure concurrently reporting more engagement (β = 0.37, *p* < 0.001), disengagement (β = 0.25, *p* < 0.001), and comfort-seeking (β = 0.15, *p* < 0.001), and less escape (β = −0.19, *p* < 0.001) to cope with academic stress. Students who reported higher grades in school reported less disengagement coping (β = −0.10, *p* < 0.05). Once all of these parent and academic factors were considered, age was associated with less disengagement coping (β = −0.11, *p* < 0.001), and (consistent with the group comparisons in Table 1) data collection timing was associated with all ways of coping except comfort-seeking.

### Longitudinal Model: Associations with Change in Ways of Coping from T1 to T2

**Fig. 2:**
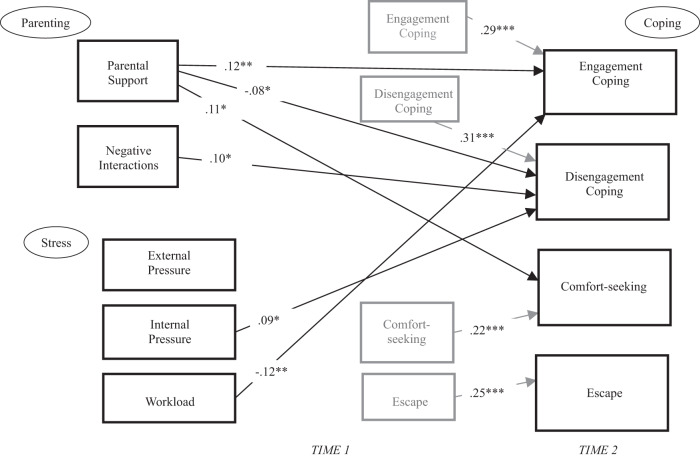
An illustration of the significant standardized path coefficients in the model of T1 and T2 measures of parenting, academic stress, ways of academic coping, and grades. *p < 0.05. **p < 0.01. ***p < 0.001. Grades in school, age, and COVID-19 data collection timing were not significantly associated with any measure of T2 coping, so they are not shown here

The results of the longitudinal model linking T1 parental support, parent negative interactions, pressures, and grades in school to the four ways of coping at T2 (adjusting for coping at T1, age, and data collection timing) are shown in Table 4 and Fig. 2. This model had a good fit to the data, χ^2^(36) = 114.39, *p* < 0.001, CFI = 0.98, RMSEA = 0.051 (90% CI 0.041 to 0.062), *p* = 0.420. Overall, the longitudinal model accounted for less variance in each way of coping relative to the concurrent model: 18% of the variance in academic engagement coping, 21% of disengagement coping, 8% of comfort-seeking, and 12% of escape.

**Table 4:** Results of Testing All Directional Paths to Academic Coping in the 1-Year Longitudinal Model (*N* = 839)

T1 Predictor	T2 Outcome	*B*	*SE B*	*p*-value	β
Engagement coping	Engagement coping	0.29	0.04	<0.001	0.29^***^
Disengagement coping	Disengagement coping	0.32	0.04	<0.001	0.31^***^
Comfort-seeking	Comfort-seeking	0.21	0.03	<0.001	0.22^***^
Escape	Escape	0.27	0.04	<0.001	0.25^***^
Parental support	Engagement coping	0.06	0.02	0.006	0.12^**^
Parental support	Disengagement coping	−0.05	0.03	0.048	−0.08^*^
Parental support	Comfort-seeking	0.09	0.04	0.010	0.11^*^
Parental support	Escape	−0.03	0.03	0.401	−0.04
Parent neg int	Engagement coping	0.00	0.02	0.987	0.00
Parent neg int	Disengagement coping	0.06	0.03	0.023	0.10^*^
Parent neg int	Comfort-seeking	0.00	0.03	0.905	0.01
Parent neg int	Escape	0.05	0.03	0.129	0.07
Ext pressure	Engagement coping	−0.01	0.02	0.484	−0.03
Ext pressure	Disengagement coping	−0.03	0.02	0.096	−0.07
Ext pressure	Comfort-seeking	0.02	0.03	0.521	0.03
Ext pressure	Escape	0.02	0.03	0.463	0.03
Int pressure	Engagement coping	0.01	0.02	0.548	0.02
Int pressure	Disengagement coping	0.05	0.02	0.018	0.09^*^
Int pressure	Comfort-seeking	0.02	0.03	0.504	0.03
Int pressure	Escape	−0.02	0.03	0.434	−0.03
Workload	Engagement coping	−0.05	0.02	0.006	−0.12^**^
Workload	Disengagement coping	0.03	0.02	0.145	0.06
Workload	Comfort-seeking	−0.04	0.03	0.176	−0.06
Workload	Escape	0.04	0.03	0.142	0.06

*Neg int* negative interactions, *Ext* external, *Int* intrapsychic. All covariances between T1 predictors, age, and data collection timing were freed if *p* < 0.10. These covariances (and correlations) are not shown in this Table, but they were similar to the results shown in Tables 1, 2, and 3. Grades in school, age, and COVID-19 timing were not significantly associated with any T2 measures in the model, so are not shown here

Model fit: χ^2^(36) = 114.39, *p* < 0.001, CFI = 0.98, RMSEA = 0.051 (90% CI 0.041 to 0.062), *p* = 0.420

*p < 0.05; **p < 0.01; ***p < 0.001

In this model, with the exception of escape, parental support was associated with better academic coping by T2, including an increase in engagement coping (β = 0.12, *p* < 0.01), a decrease in disengagement coping (β = −0.08, *p* < 0.05), and an increase in comfort-seeking (β = 0.11, *p* < 0.01; see Table 4 and Fig. 2). Negative interactions with parents also played role, as this measure was associated with increases in disengagement coping by T2 (β = 0.10, *p* < 0.05). In addition, there were some significant directional paths from intrapsychic pressure and workload pressure, with the former associated with increases in T2 disengagement coping (β = 0.09, *p* < 0.05), and the latter associated with decreases in T2 engagement coping (β = −0.12, *p* < 0.01).

## Discussion

Although suggested in numerous developmental and social theories of social relationships, stress, and coping (e.g., Skinner & Edge, 2002b), there has been little investigation of whether adolescents’ experiences of parental support and negative parent-adolescent interactions relate to their engagement and disengagement ways of coping with academic stress. In general, the findings support the conclusion that more support from parents and fewer negative parent-child interactions are positive for adolescents’ concurrent and future reliance on more engagement and less disengagement ways of coping with academic stressors, before and after considering the significant contributions to coping found for academic pressures and achievement (i.e., grades in school), and controlling for age and COVID-19 data collection timing.

### Parents, Academic Workload and External Pressures, and Coping

Past research has found that many adolescents respond to academic stress with what are usually constructive and useful coping responses, such as strategizing, problem-solving, and support- or help-seeking (e.g., Skinner & Saxton, 2019). Yet, the findings of the present study provide evidence that this may be shaped by parents, consistent with decades of research showing that parents and families play crucial roles in students’ academic well-being (see Barger et al., 2019 for a review). In a concurrent (T1) multivariate path model, adolescents who reported more parental support reported more engagement coping and comfort-seeking, whereas those who reported more negative interactions (experiences of rejection and coercion) with parents relied more on disengagement and escape to cope with academic stress. Interestingly, there was no evidence that support from parents was associated with a reduction in adolescents’ reliance on negative ways of coping (i.e., less disengagement and escape ways of coping), or that more negative interactions between adolescents and their parents undermined the use of positive ways of coping (i.e., less engagement and comfort-seeking ways of coping). In a parallel model examining one-year longitudinal changes in coping, parental support (relative to negative interactions) had slightly more far-reaching associations with coping – adolescents who reported more support increased in both constructive academic coping (i.e., an increase in engagement and comfort-seeking ways of coping) and also decreased in disengagement coping by T2. Conversely, adolescents who reported more negative interactions with parents showed higher levels of disengagement coping by T2. As suggested in SDT, the provision of support (and fewer experiences of rejection and coercion) by parents could be meeting adolescents’ needs for relatedness, competence, and autonomy, while also providing a source of helpful advice and comfort for academic stress. By meeting adolescents’ needs, parents could be seen as a good source of support to deal with stress outside the home, while also helping adolescents feel engaged and autonomous in their choice of daily activities and competence in facing stressful events (Raftery-Helmer & Grolnick, 2015). They could also provide some respite from academic stress because supportive relationships are enjoyable and distracting (e.g., positive mood is associated with more academic engagement and motivation; Klootwijk et al., 2022).

The findings of the present study also indicate that academic stress, measured as pressures due to workload, external demands for better performance by parents and teachers, and intrapsychic desires for high achievement, also relate to how adolescents report they cope, both concurrently and (although less so) longitudinally, perhaps because academic pressures are more specific to the current year’s situation. First, regarding workload pressure, students who reported more pressure were found to concurrently report less reliance on positive ways of coping – they used fewer engagement strategies (e.g., strategizing and commitment/planning), used more disengagement (e.g., more concealment, self-pity, and rumination), and they engaged in more cognitive strategies to minimize the importance of academic outcomes as a way of dealing with their academic stress. In the longitudinal model, workload pressure also foreshadowed a decline in engagement coping by T2. Second, external pressure was also problematic for coping, given that it was significantly associated with more use of escape. Thus, although there are very few longitudinal (or intensive repeated measures) studies on the topic of academic stressors and coping over time, these findings and those of others (e.g., Iida et al., 2017) suggest that feelings of excessive workload pressures, and to a lesser extent external pressure for academic performance, covary with poorer ways of coping and (despite what could be the good intentions of parents and teachers who try to encourage achievement by applying some external pressure) can result in declines in engagement coping over time. Such interrelations reveal potential risk for a negative spiral of being overwhelmed by academic pressures, more external pressure, and poorer coping responses feeding into each other as they unfold over time.

### Academic Intrapsychic Stress and Coping

Although there were modest positive correlations between academic workload pressure, external pressure to perform, and intrapsychic pressure, which were consistent with, but slightly weaker than, past research (Sun et al., 2011), the analyses in the present study revealed a few similarities and multiple differences between the findings for academic stress in the form of intrapsychic pressure compared to workload and external pressures. First, with regards to similarity with the findings for workload and external pressures, adolescents who reported more intrapsychic pressure to achieve reported more disengagement coping – they were more likely to ruminate, conceal, and engage in self-pity. Thus, as found for workload and external stress, higher self-expectations are indicative of some problem ways of coping.

Second, intrapsychic pressure, although stressful and associated with some coping concerns, seems to covary with signs of more behavioral engagement with academic work. Different to workload pressure, intrapsychic pressure was associated with more engagement coping, more comfort-seeking, and less escape. Thus, although good for active approach behaviors, intrapsychic stress can potentially come with emotional and cognitive costs (e.g., more rumination). It is likely that high stress due to intrapsychic pressure characterizes adolescents who highly value doing well at school and may need opportunities to “switch off” to keep their personal expectations from becoming detrimental to their emotional or academic well-being, which otherwise could lead to lower performance and/or burnout (Vizoso et al., 2019). In fact, the longitudinal analyses did show that adolescents who reported more intrapsychic pressure were higher in disengagement coping by T2. Thus, such stress due to intrapsychic pressure for high achievement may signal risk and this complex pattern of associations may be indicative of future problems. For example, in one 3-year longitudinal study, academic stress combined with high expectations in the early adolescent years was associated with lower academic performance three years later in high school (Kaplan et al., 2005). A similar pattern of effects has been seen in studies examining the effects on coping of internal pressure in the form of introjected self-regulation (Skinner & Saxton, 2019).

### Associations with Age

There was mixed evidence for associations of age with parenting, academic pressures, and coping. In the zero-order correlations, older adolescents seemed to show signs of more problems at home and at school – they reported poorer relationships with parents, more academic stress, less engagement coping, and more escape. However, in the multivariate analyses, age only remained significantly correlated with one other measure, and in the opposite direction, showing that age was associated with *less* disengagement coping. The findings of this study are generally consistent with other research showing the increasing academic pressures that occur as adolescents get older (e.g., Pascoe et al., 2020), but the findings extend on this past research identifying that the associations of age with academic coping that have been reported (Ben-Eliyahu & Kaplan, 2015) may be reduced substantially after adjusting for parenting support and negative interactions and academic stressors, suggesting that these processes may account for some of the age differences or changes in coping.

### Effects of COVID-19

Regarding COVID-19 and the timing of the study, data were collected during three periods: before the start of the first nationwide stay-at-home orders in the country, during the stay-at-home orders, and after return to in-class learning. Adolescents who completed surveys after returning to in-class learning (and who were the oldest on average) stood out as having both more problems and using a mixed pattern of coping; they reported the poorest relationships with parents, the highest levels of workload and external pressure, and the most escape to cope with stress, but also reported the best grades, the most engagement coping, and the least disengagement coping. It is also worth mentioning that parental support was highest, parental rejection was lowest, and workload and external pressures, grades in school, and engagement coping were lowest during stay-at-home orders. These findings are consistent with anecdotal reports from families in the region about the positive family relationships and the reduced academic workloads experienced during the lockdown. The main period of K-12 remote learning in the region was expected to be (and was) relatively short (about one month; Australian Institute for Teaching and Learning, 2021). At this time, there were few cases of COVID-19 circulating in Australia (especially in the region where this study was conducted), people were allowed outdoor time together in family units each day, and many families received federal financial support to help them adjust to staying at home. Yet, schools were impacted; they had to quickly move to online learning, which meant changing methods of direct instruction, less scaffolding of individual student learning, and changing assessment and feedback practices. These changes had flow-through effects into the rest of the 2020 school year (e.g., attendance of students declined; standardized achievement exams were canceled; Australian Institute for Teaching and Learning, 2021). This seems to have led to great variability in educational strategies during this time, but studies also suggest there was little *overall* impact on student achievement (but the engagement and achievement of the most disadvantaged students may have been adversely affected; Gore et al., 2021). However, in the present study, COVID-19 timing was confounded with survey format (in-person vs. online) and was associated with adolescents’ age. Thus, the analyses of COVID-19 timing were a way to control for this as a potential confound rather than directly addressing how COVID-19 lockdown may have impacted on parent-adolescent relationships or academic coping.

### Study Strengths and Limitations

This study had multiple strengths including a large sample, and good gender and racial/ethnic diversity, measurement of three forms of academic stress and many of the most common ways of academic coping found among adolescents, and a focus on parents as social foundations and impediments to coping in an important adolescent functional domain of academics. Nevertheless, there are three limitations that could be addressed when designing future research. First, this was a convenience sample drawn from the first schools to express interest in study participation. Future research is needed to determine whether the findings are generalizable to other schools, regions or nations. Second, stress, coping, and relationships were measured using adolescent self-report. Although self-reports of stress and coping are likely some of the best tools for understanding these experiences for adolescents, it may be that the intensity of academic stress affects reports of perceived coping in other ways. Also, relationship qualities could be measured by drawing on parents’ reports to corroborate and extend on the current analyses. Self-report measures of relationship support and rejection do not always highly covary with reports from others (De Los Reyes et al., 2019).

Third, although the study design was longitudinal with a good retention rate, only two waves of data were collected. Given the support for the hypothesis that parenting predicts changes in adolescent coping, this paves the way for including more repeated assessments. A higher number of assessments would allow an analysis of patterns of change in stress and coping across multiple years. Repeated measures (or experimental) research, or even carefully designed intervention research (Frydenberg, 2018), could clarify some of the possibilities regarding directions of associations, pathways, and processes that unfold over time that could not be answered with the current study design. For example, adolescents’ level of academic stress and their ways of coping have the potential to change relationships with parents, alongside relationships having an impact on academic stress and coping; parents may become more supportive when they see their children struggling with academic workload, or parents may become more rejecting and coercive when their children conceal and engage in self-pity. Furthermore, family commitments and structure, such as work commitments, the presence of another caregiver, and the number of siblings, could be important to parents’ availability and to children’s coping and academic experiences. Also, as previously mentioned, coping can reduce or even exacerbate stress at the same time that different stress levels prompt particular ways of coping. All of these questions would be enriched by considering mediators and mechanisms that more precisely identify what it is about social resources or problems that explain adolescents’ ways of coping with academic stress. Studies that consider the developmental dynamics among these processes, including both feed-forward effects from parents to adolescent stress and coping as well as feedback effects from adolescent stress and coping to changes in parenting, would be especially useful (Skinner & Edge, 2002a).

### Implications for Future Research and Practice

In addition to the future research directions that were suggested in the previous section, there are two additional considerations from the current findings that yield future research ideas. First, some ways of coping can be constrained by the context and opportunities available to put in place coping actions – for example, comfort-seeking may only be possible when adolescents have parents who are more emotionally and physically available. Thus, there is more to do to understand the social foundation of coping. Second, coping was measured as if it was static and comes in independent units – instead coping with stress has been described as complex, time-varying, and dependent on the changing contextual demands (Skinner & Zimmer-Gembeck, 2016). It could be that the overall configuration of coping responses at any one time or the pattern of coping over time can be as (or more) important to capture than the use of any one way of coping (Masters et al., 2023). For example, low comfort-seeking coping may be an effective and beneficial way of coping with workload stress, but only when strategizing is high. Further, disengagement may be a nonproductive response to stress regardless of whether other ways of coping are used. Thus, the focus on each way of coping as separate from others in the present study may have missed important coping profiles or repertoires that could be even more strongly related to parent and peer relationships or with academic stress due to workload or self-expectations.

Regarding application of the findings to practice, school-based or other programs designed to help adolescents cope more productively with academic stressors, whether stress comes from perceived workload pressure or because of personal expectations of high achievement, should address how academic life can be supported by family life. In addition, not all academic stress yields the same pattern of coping, and this could be addressed more precisely in support programs (as well as in future research). Most notably, workload and external pressures were associated with less engaged and more disengaged ways of coping, consistent with what would be expected for possible uncontrollable forms of stress. In contrast, intrapsychic performance pressure had associations with positive, approach-type coping strategies but also was associated with more disengagement, that also increased over time. Overall, the most productive approach to assist a student to constructively cope with academic stress could depend on the type of pressure most prominent for that individual student. Those designing programs to help adolescents cope with academic stress should keep these differential patterns in mind in order to address such individual needs.

## Conclusion

The environment parents provide, and their modeling and socialization of coping, are often described as foundations for the development of their children’s ways of coping with stress. This implies that parental support and negative interactions with parents should be social foundations for adolescents’ ways of coping with workload and other *academic* stressors they experience. However, this possibility had received little research attention, leaving a gap in knowledge of whether parent-adolescent relationships spill over into adolescents’ academic coping. The aim of this study was to determine if parental support and negative parent-adolescent interactions were associated with adolescents’ engagement and disengagement ways of coping with academic stress (concurrently and over one year), considering stress from workload, intrapsychic pressure, and external pressure to perform. The findings showed that, above and beyond the many ways that workload, intrapsychic, and external academic pressures and adolescents’ achievement related to ways of academic coping (as well as controlling for adolescents’ age and COVID-19 timing of the study), good parental relationships are positive for adolescents’ concurrent and future reliance on more engagement and less disengagement ways of coping with academic stressors. Adolescents who report more parental support report more use of engagement ways of coping, such as strategizing and help-seeking, and more comfort-seeking. Adolescents who report more negative interactions with their parents report more use of disengagement ways of coping, such as rumination and concealment, and escape. These findings confirm decades of research demonstrating that parents are connected to adolescents’ academic lives, but also expands on this past research to suggest that parents play a unique role in how their adolescents cope with a range of academic stressors.
